# Characterization of the different S6Ks isoforms interacting partners

**DOI:** 10.1186/2049-3002-2-S1-P68

**Published:** 2014-05-28

**Authors:** Isadora Pavan, Flávia Ferreira, Lidia de Freitas, Mariana Tavares, Camila do Amaral, Fernando Simabuco

**Affiliations:** 1 University of Campinas, Limeira, Brazil

## 

mTOR signaling pathway has been related to several human diseases and disorders, including obesity, diabetes and many types of cancer. In the cell, mTOR signaling is responsible to induce cell growth and metabolism upon activation by signals from nutrients processing, such as insulin, ATP and amino acids. S6Ks, which phosphorylates ribosomal S6 protein, are effectors of the mTOR pathway. S6K family is composed of two genes (encoding S6K1 and S6K2) and different isoforms from alternative translation initiation (p70-S6K1, p85-S6K1, p54-S6K2, p56S6K2) and alternative splicing (p31-S6K1). The main function of S6Ks, upon activation, is to lead increased protein synthesis. Nevertheless, one of the main questions in this field is to address the specific roles of the different S6Ks isoforms. In this study, we overexpressed p70-S6K1, p85-S6K1 and p54-S6K2 in human cells and analyzed by immunoprecipitation, followed by mass spectrometry identification, the different interacting partners of those S6Ks isoforms. The different roles of S6Ks isoforms were also evaluated in an adipocyte model and in cancer cell lines. Our study provide new possible functions to the different S6Ks isoforms which may help the understanding of their roles in the mTOR signaling pathway, metabolism and cancer.

